# The N-Terminus of the HIV-1 p6 Gag Protein Regulates Susceptibility to Degradation by IDE

**DOI:** 10.3390/v10120710

**Published:** 2018-12-12

**Authors:** Adrian Schmalen, Julia Karius-Fischer, Pia Rauch, Christian Setz, Klaus Korn, Petra Henklein, Torgils Fossen, Ulrich Schubert

**Affiliations:** 1 Institute of Virology, Friedrich-Alexander-University Erlangen-Nürnberg (FAU), 91054 Erlangen, Germany; Adrian.Schmalen@uk-erlangen.de (A.S.); julia.karius@fau.de (J.K.-F.); Pia.Rauch@uk-erlangen.de (P.R.); Christian.Setz@uk-erlangen.de (C.S.); Klaus.Korn@uk-erlangen.de (K.K.); 2 Institute of Biochemistry, Charité—Universitätsmedizin Berlin, 10117 Berlin, Germany; petra.henklein@charite.de; 3 Department of Chemistry, University of Bergen, 5020 Bergen, Norway; Torgils.Fossen@uib.no

**Keywords:** HIV-1, p6, insulin-degrading enzyme, L1P, L449P, cleavage-site, degradation, proteolysis, stability, subtypes

## Abstract

As part of the Pr55^Gag^ polyprotein, p6 fulfills an essential role in the late steps of the replication cycle. However, almost nothing is known about the functions of the mature HIV-1 p6 protein. Recently, we showed that p6 is a bona fide substrate of the insulin-degrading enzyme (IDE), a ubiquitously expressed zinc metalloprotease. This phenomenon appears to be specific for HIV-1, since p6 homologs of HIV-2, SIV and EIAV were IDE-insensitive. Furthermore, abrogation of the IDE-mediated degradation of p6 reduces the replication capacity of HIV-1 in an Env-dependent manner. However, it remained unclear to which extent the IDE mediated degradation is phylogenetically conserved among HIV-1. Here, we describe two HIV-1 isolates with IDE resistant p6 proteins. Sequence comparison allowed deducing one single amino acid regulating IDE sensitivity of p6. Exchanging the N-terminal leucine residue of p6 derived from the IDE sensitive isolate HIV-1_NL4-3_ with proline enhances its stability, while replacing Pro-1 of p6 from the IDE insensitive isolate SG3 with leucine restores susceptibility towards IDE. Phylogenetic analyses of this natural polymorphism revealed that the N-terminal leucine is characteristic for p6 derived from HIV-1 group M except for subtype A, which predominantly expresses p6 with an N-terminal proline. Consequently, p6 peptides derived from subtype A are not degraded by IDE. Thus, IDE mediated degradation of p6 is specific for HIV-1 group M isolates and not occasionally distributed among HIV-1.

## 1. Introduction

Within Gag, the HIV-1 p6 protein is synthesized as the C-terminal domain [1]. It consists of only 52 amino acids (aa), thereby belonging to the smallest lentiviral proteins. After autocatalytic activation of the viral protease and concomitant to budding of immature viral particles, it is released from the Pr55^Gag^ precursor during virus morphogenesis [2,3,4]. Pivotal roles during the late steps of the viral replication cycle have been described to be regulated by the p6 domain of Gag. By its two Late (L-) domains, it regulates the interaction of Gag with components of the cellular endosomal sorting complex required for transport (ESCRT). Thereby, the PT/SAP motif binds to the tumor susceptibility gene 101 (Tsg101), while YP(X)_n_L recruits the apoptosis-linked gene 2-interacting protein X (ALIX) [5,6,7,8]. Eventually, these proteins will recruit further ESCRT components, resulting in the final abscission of budding virions from the plasma membrane [9]. The original function of the ESCRT-machinery is the regulation of topologically equivalent membrane fission events during membrane protein trafficking and cytokinesis [10,11,12]. Deletion of the PTAP L-domain in p6 results in a severe budding defect that can be rescued by overexpression of ALIX [5,6,7,8,9]. In contrast, mutation of the ALIX-binding site has less prominent implications for the budding virion [5,6,13]. Furthermore, the incorporation of Vpr into budding virions is mediated by the p6 protein [14,15].

Hitherto, all known functions ascribed to p6 during assembly, release, and maturation of progeny virions take place with p6 being the C-terminal part of the Gag precursor. However, the role of free p6, either as part of a mature virion infecting a new host cell or stemming from decayed virions in the extracellular space has not been investigated yet. Recently, we were able to show that the mature HIV-1 p6 protein is a bona fide substrate for a cellular protease, the insulin-degrading enzyme (IDE) [16]. IDE is a 120 kDa highly conserved zinc-metalloprotease with homologs in plants, fungi and even bacteria [17,18,19,20,21]. In humans, it is involved in the degradation of short peptide hormones like its eponymous substrate insulin and the clearance of amyloidogenic peptides like amyloid β [22,23]. Furthermore, IDE is ubiquitously expressed in human tissues and cells, including CD4^+^ T cells and macrophages [18,24,25,26]. Stabilization of p6 under certain conditions impaired the replication capacity of HIV-1 in an Env-dependent manner, indicating a potential function of p6 that exceeds its hitherto described L-domain function [16].

We were able to show that this phenomenon appears to be specific for HIV-1, since none of the tested p6 sequences from HIV-2 and SIV, nor the p6 homolog from EIAV, p9, were degraded by IDE [16]. However, the phylogenetic background of the degradation of HIV-1 p6 by IDE has not been investigated thoroughly. Notably, it is unclear to which extent the degradation of p6 is conserved within HIV-1.

In the phylogenetic history of HIV-1, four transmission events across the species barrier of SIV to humans have been identified, each leading to a distinct group of HIV-1 [27,28,29,30,31]. However, only the HIV-1 group M established a worldwide pandemic, while the other groups are mostly locally distributed [29,31,32]. Group M can be further classified into distinct subtypes, characterized by their genetic relation. While HIV-1 isolates from the same subtype can genetically differ in 10% to 20% to a reference sequence, the intersubtype genetic variation is usually 20% to 35%, depending on the genetic region compared [33]. The formation of circulating recombinant forms (CRF), resulting from recombination events in superinfected patients, further increases the complex subtype diversity of HIV-1 group M [34,35,36].

Here, we describe two HIV-1 isolates that contain IDE-insensitive p6 variants. Sequence comparison of those p6 proteins allowed us to deduce one single aa substitution that impairs degradation of p6 by IDE, namely proline-1 (Pro-1) at the N-terminus of p6. Replacement of the leucine residue at position one of p6 derived from the IDE sensitive HIV-1_NL4-3_ by a proline impairs IDE-dependent degradation of p6. Vice versa, substitution of the proline at position one of the IDE-insensitive p6 variant derived from HIV-1 SG3 by a leucine residue restores the susceptibility towards IDE. In contrast to our previously generated IDE-insensitive p6 mutant, this substitution represents a naturally occurring polymorphism of HIV-1 p6 [16,37].

The HIV-1 p6 L1P mutant mostly behaves like previously described IDE insensitive p6 mutants regarding virus release, Gag processing to p24 and replication capacity. This particular N-terminal aa grants insight in the phylogenetic background and the extent of the p6 IDE interaction in HIV-1. The N-terminal leucine is characteristic for HIV-1 group M, since HIV-2, SIV of sooty mangabeys (SIVsm) and the other HIV-1 groups have a proline or a heterogenic N-terminus at position one of p6. Intriguingly, the HIV-1 subtype A has restored the IDE-insensitive p6 by exclusively expressing p6 variants with an N-terminal proline. Our data so far indicate that the susceptibility of p6 towards IDE-mediated degradation was phylogenetically formed alongside the leucine residue at position one of p6, which is characteristic for most non-A subtypes of HIV-1 group M.

## 2. Materials and Methods

### 2.1. Cell Culture and Transfection

HeLa, HeLa TZMbl wild type (*wt)*/IDE KO and HEK293T cells were maintained in DMEM containing 10% (*v*/*v*) inactivated fetal calf serum (FCS), 2 mM l-glutamine, 100 U/mL penicillin and 100 μg/mL streptomycin. CEMx174 M7 R5 cells and peripheral blood mononuclear cells (PBMC) were maintained in RPMI medium 1640 containing 10% (*v*/*v*) FCS, 2 mM L-glutamine, 100 U/mL penicillin and 100 µg/mL streptomycin. All cell culture media and reagents were purchased from Gibco (Life Technologies, Carlsbad, CA, USA).

Cells were transfected with Lipofectamine 2000 (Thermo Fisher Scientific, Waltham, MA, USA) according to the manufacturer’s protocol. Then, 24 h post transfection cells were lysed in RIPA buffer (150 mM NaCl, 50 mM Tris-HCl pH 8.0, 1% NP-40, 0.5% Na-deoxycholate, 0.1% SDS, 10 mM EDTA) containing 5 mM N-ethylmaleimide (NEM), and 1 mM phenylmethylsulfonylfluoride (PMSF).

### 2.2. HIV-1 Isolates and Expression Plasmids

The proviral HIV-1_NL4-3_ and HIV-1 SG3 expression constructs have been described elsewhere [38,39]. The HIV-1_NL4-3_ derived expression construct pNL*env*1 *wt* has been described previously [40]. The isolates 93BR020, 99ET_14, 92UG024, VI557, 92UG029 and 00KE_KER2008 are isolates of various HIV-1 group M subtypes obtained from the National Institute for Biological Standards and Control (NIBSC, Ridge, Herts, UK). Isolate 4lig7 is a multidrug-resistant recombinant virus of HIV-1 subtype B that has been generated in the diagnostic laboratory of the Institute of Clinical and Molecular Virology of the Universitätsklinikum Erlangen (Erlangen, Bavaria, Germany) [41].

Mutations of p6 were inserted by site-directed mutagenesis PCR (QuikChange Lightning, Agilent Technologies, Santa Clara, CA, USA) using a pair of complementary primers. Thereby, the pNL*env*1 L1P and HIV-1_NL4-3_ L1P mutants were generated using the primer 5’-gg cca ggg aat ttt ccg cag agc aga cca gag cc-3’ and its corresponding reverse complement, whereas the mutant SG3 P1L was obtained using the primer 5’-gga cca ggg aat ttt ctg cag agc aga cta gag cc-3’ and its corresponding reverse complement.

### 2.3. Virus and VLP Isolation

Virus or virus-like particles (VLP) containing cell culture supernatant was harvested 48 h after transfection of 293T cells and passed through a 0.45 μm pore-size filter. Alternatively, CEMx174 M7 R5 cells were infected with cell culture supernatant containing infectious HIV-1 particles. Infected cells were provided with 5 × 10^6^ uninfected CEMx174 M7 R5 cells along with fresh medium on day two and four post infection to increase virus yield. After syncytium formation or seven days post infection (dpi), the supernatant was harvested and passed through a 0.45 μm pore-size filter. The viral particles were pelleted through a 20% (*w*/*v*) sucrose cushion (20,000× *g*, 4 °C, 90 min) and stocks were normalized for p24, as quantified by p24 ELISA (Aalto Bio Reagents LTD, Rathfarnham Village, Dublin, Ireland).

### 2.4. Infection of Cells

Replication assays were performed as reported previously [16]. In short, PBMCs were isolated from buffy coats of several blood donors and stimulated with phytohaemagglutinin (PHA-P) and Interleukin 2 (IL-2) for three days. Then, 1 × 10^6^ PBMCs were incubated overnight with virus preparations equivalent to 0.031 ng of p24 for HIV-1_NL4-3_ or 0.016 ng of p24 for HIV-1_SG3_, respectively and cell culture supernatants were collected every second or third-day post infection (dpi). Where indicated, the cells were treated with 10 μM 6bk (Phoenix Pharmaceuticals, Burlingame, CA, USA), and, as part of the medium change on the designated dpi, fresh 6bk was added. Virus replication was assessed by quantification of the virus-associated RT activity by [^32^P]-TTP incorporation, using an oligo(dT)-poly(A) template as described elsewhere [42].

The respective replication profiles were depicted as a diagram (y-axis: RT activity; x-axis: dpi) and the area under the curve was calculated and defined as the replication capacity. In every experiment, the replication capacity of the untreated cells was set to 100% and compared with the indicated variants.

### 2.5. SDS PAGE and Western Blotting

Protein samples were separated by SDS-PAGE [43] and subsequently transferred onto 0.2 µm nitrocellulose (NC) membranes (GE Healthcare, Chicago, IL, USA). Membranes were fixed with 4% (*w*/*v*) paraformaldehyde (PFA). Subsequently, fixation was stopped by washing with 1 M glycine in PBS, followed by blocking with 3% (*w*/*v*) bovine serum albumin (BSA) supplemented with 0.1% sodium azide. Membranes were incubated with the appropriate primary antibody (Ab) in 1% BSA. Gag was detected using a rabbit antiserum recognizing p24 (Seramun Diagnostica GmbH, Heidesee, Brandenburg, Germany). The HIV-1 p6-specific antiserum (Seramun Diagnostica GmbH) has been described earlier [44]. The anti-rabbit secondary Ab coupled to HRP was obtained from Dianova (Dianova GmbH, Hamburg, HH, Germany) and diluted 1:10,000 in 5% nonfat dry milk/PBS-T. In some experiments, the amount of protein blotted on NC membranes was densitometrically quantified with the software AIDA (Elysia Raytest GmbH, Angleur, Walloon, Belgium).

### 2.6. In Vitro Degradation Assay

For production of S10, confluent dishes with HeLa cells were extensively washed, cells were harvested and subsequently lysed with IDE enzyme buffer (150 mM NaCl, 50 mM Tris pH 7.4, 0.5% (*v*/*v*) Triton X-100). Debris and nuclei were removed by centrifugation at 10,000× *g* for 10 min. Protein content was determined by BCA-Assay (Pierce™, Thermo Fisher Scientific, Waltham, MA, USA) and aliquots stored at −80 °C.

For the in vitro degradation of viral p6 (*v*p6), VLPs were pelleted and lysed in IDE enzyme buffer. Subsequently, 5 µg of S10 were incubated with the virus lysate containing *v*p6 at 37 °C. Reactions were stopped at the time points indicated by heat inactivation at 95 °C and addition of sodium dodecyl sulfate (SDS) sample buffer. Remaining p6 was detected by Western blotting using an anti-p6 rabbit antiserum [44]. Experiments carried out with recombinant IDE (rIDE, Calbiochem, Merck Millipore, Burlington, MA, USA) were performed in a HEPES-based IDE enzyme buffer (150 mM NaCl, 50 mM HEPES pH 7.4, 0.5% (*v*/*v*) Triton X-100).

### 2.7. Peptide Synthesis and Mass Spectrometry

The synthesis of the peptide was performed on a CEM Microwave Peptide Synthesizer Liberty 1 on a 0.1 mM scale with 125 mg H-Gln(Trt) Hmpb-Chematrix-resin (capacity 0.47 mmol/g; PCAS BioMatrix Inc, St-Jean-sur-Richelieu, QC, Canada) or 100 mg Gln-TCP resin (capacity 0.5 mmol/g, Intavis, Köln, K, Germany) using the Fmoc-strategy (N-(9-fluorenyl)methoxycarbonyl). Couplings were carried out with N, N, N′, N′-Tetramethyl-O-(1H-benzotriazole-1-yl)uronium hexafluoro-phosphate (HBTU) in N-methylpyrrolidone as coupling agent by a temperature of 50 °C. Deprotection of the Fmoc group was performed during the complete synthesis with 5% Piperazine, 0.1 M 1-Hydroxybenzotriazole (HOBt) in N, N-dimethylformamide. The final deprotection from the resin was performed with 95% TFA in water containing 3% triisopropylsilane. The crude peptide was purified by reverse phase HPLC on a 10 μm Phenomenex Gemini C18 column (21.2 × 250 mm, 110 Å) with a linear gradient of 40% A to 65% B in 50 min (A: 1000 mL water, 2 mL TFA; B: 500 mL acetonitrile, 100 mL water, 1 mL TFA) at a flow rate of 15 mL min-1 with spectrophotometric monitoring at λ = 220 nm. The fractions were checked by RP-HPLC (Shimadzu LC10) on a Zorbax 300SB C18 column (4.6 × 250 mm, 5 μ, 300 Å) with a linear gradient of 10% to 100% B over 45 min and mass spectrometry (Voyager DE PRO MALDI-TOF mass spectrometer, Applied Biosystems, Foster City, CA, USA, linear mode) [16].

### 2.8. In Silico Analysis

Sequence alignments were generated using the Clustal Omega multiple sequence alignment software [45,46,47]. For the phylogenetic tree analysis, all genome sequences from the HIV-1/SIVcpz compendium 2016 and the HIV-2/SIVsm compendium 2015 were collected and merged [48,49]. Circulating recombinant forms of HIV-1/SIVcpz were removed. Furthermore, SIVcpz sequences closely related to HIV-1 group M were added manually to the sequence collection. Namely, the inserted sequences (with the corresponding Genbank accession number) were derived from the SIVcpz isolates MB897 (EF535994), MB66 (DQ373063), LB7 (DQ373064), EK505 (DQ373065), MT145 (DQ373066), CAM3 (DQ373065), CAM5 (DQ373065), CAM13 (AY169968), GAB1 (X52154), GAB2 (AF382828) and DP943 (EF535993) [28,31]. Subsequently, a phylogenetic tree was generated using the software package SATé© version 2.2.7 for Windows [50,51,52]. Visualization and processing of the phylogenetic tree were conducted with the software FigTree version 1.4.3 [53]. The phylogenetic tree for HIV-1 and HIV-2 was generated in circular form as cladogram with a mid-point root using whole genome DNA sequences. The isolates have been colored with respect to the first aa of the p6 protein.

The first codon of the p6 protein from the HIV-1/SIVcpz and HIV-2/ SIVsm filtered web-alignment 2017 has been collected and translated into the corresponding aa [54]. The aa occurrences at position one of the p6 protein for the HIV-1 groups and subtypes, and for HIV-2 as well as for SIVsm were quantified.

Protein structure prediction was performed using the Phyre2 protein folding recognition server [55]. Visualization of the .pdb-files was carried out using the software PyMOL [56].

### 2.9. Ethical Statement

The study was conducted in accordance with the Declaration of Helsinki, and the protocol was approved by the Ethics Committee of the Medical Faculty of the Friedrich-Alexander University Erlangen-Nürnberg (Project identification code: 3761; 2 March 2017).

## 3. Results

### 3.1. p6 Derived from the HIV-1 Isolates SG3 and 4lig7 is not Degraded by IDE

Upon virus entry, p6 becomes highly susceptible to IDE mediated degradation. Previously, we reported that at least 12 different p6 variants of HIV-1 are a substrate of IDE. In contrast, neither one of the investigated p6 proteins derived from HIV-2 or SIV, nor the EIAV homolog p9 were degraded by IDE [16]. To investigate, to which extent this phenomenon is conserved within HIV-1, we further analyzed the susceptibility of different p6 variants of HIV-1 to IDE mediated degradation.

To analyze the susceptibility of p6 derived from various isolates to IDE mediated degradation, we performed an in vitro degradation assay using a cytosolic HeLa cell extract (S10) [16]. One of the tested isolates is SG3, an X4-tropic molecular HIV-1 clone of the subtype B with a pronounced cytopathic effect in human and chimpanzee lymphocytes [40]. Furthermore, we analyzed the stability of p6 derived from the X4-tropic HIV-1 field isolate 4lig7. This isolate also belongs to the subtype B and is known for several resistance mutations within the reverse transcriptase (RT) and the protease (PR) open reading frame, causing resistance to all nucleoside RT inhibitors and to most PR inhibitors [57].

**Figure 1:**
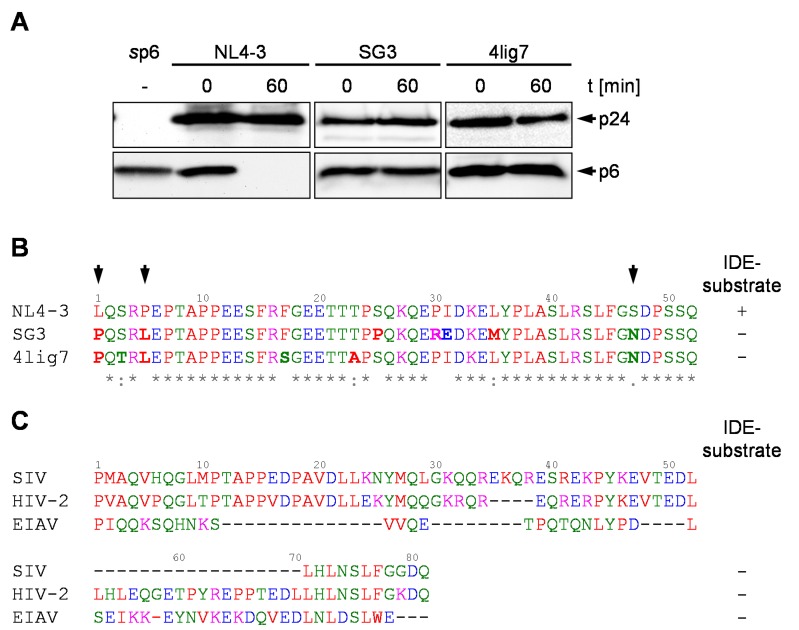
p6 derived from the HIV-1 isolates SG3 and 4lig7 is not degraded by IDE. (**A**) *v*p6 derived from NL4-3, SG3 or 4lig7 was incubated with S10 extract. p6 and p24 probings were detected by Western blotting. *s*p6 served as staining control. Representative Western blots of three independent experiments are shown. (**B**) Alignment of the p6 aa sequences of HIV-1 NL4-3, SG3, and 4lig7. Aa that differ in the sequence of SG3 and 4lig7 compared to NL4-3 are written in bold. Common polymorphisms of SG3 and 4lig7 are highlighted by arrows. Conserved aa positions in the sequence alignment are indicated by asterisks. Furthermore, aa exchanges between strongly (colon) and weakly (dot) similar aa residues are specified [45,46,47]. (**C**) Sequence alignment of IDE insensitive p6 peptides from HIV-2, SIV and EIAV p9 peptide [16]. The sequence of HIV-2 p6 originates from the isolate ROD10, SIV p6 from SIVmac239, and EIAV p9 from the isolate EIAV_Wyoming_ [16,58]. Colors of the sequence alignments according to the physicochemical properties of the aa, as proposed by the Clustal Omega multiple sequence alignment software [45,46,47].

To obtain sufficient amounts of viral p6 (*v*p6) isolated from released virions, HEK293T cells were transiently transfected with the proviral expression construct SG3 and, as a control, the subgenomic HIV-1 expression plasmid pNL*env*1, representing an *env*-deleted version of pNL4-3 [16,38,39]. Since no molecular clone of 4lig7 is available, CEM cells were infected with 4lig7 and the released virions were isolated. Viral particles were lysed in IDE buffer and incubated with S10 at 37°C for the times indicated. Surprisingly, *v*p6 proteins derived from HIV-1 SG3 or 4lig7, respectively, were completely stable, while the positive control, *v*p6 derived from HIV-1_NL4-3_, was entirely degraded within 60 min (Figure 1A). Sequence comparison of the p6 peptides revealed that the SG3 p6 peptide differs in seven aa and 4lig7 in six aa compared to the sequence of HIV-1_NL4-3_ p6. Furthermore, SG3 and 4lig7 share three aa polymorphisms not present in the sequence of HIV-1_NL4-3_, namely the proline residue at position one, leucine residue at position five and the asparagine residue at position 47 of p6 (Figure 1B). Interestingly, also the IDE-insensitive p6 proteins of HIV-2 and SIVmac239 and the p9 protein of EIAV contain an N-terminal proline residue, indicating that the presence of an N-terminal proline might correlate with insensibility of p6 to IDE-mediated degradation (Figure 1C).

### 3.2. Proline at the N-Terminus of p6 Prevents IDE-Mediated Degradation

**Figure 2:**
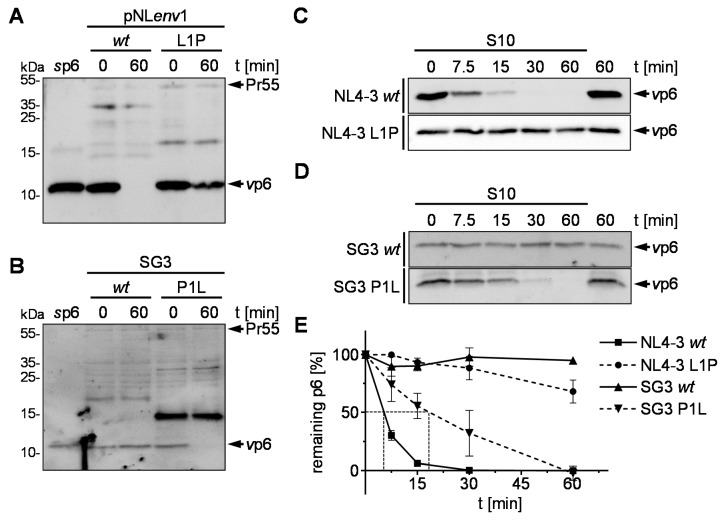
Pro-1 in p6 impairs IDE-mediated degradation. Lysates of VLPs derived from pNLenv1 *wt* and L1P (**A**) or viral particles of SG3 *wt* and P1L (**B**) were incubated with S10. Reactions were stopped by heat inactivation either immediately or after incubation for 60 min at 37 °C. Representative Western blots of three independent experiments are shown. *v*p6 derived from NL4-3 *wt* and L1P (**C**) or SG3 *wt* and P1L (**D**) were incubated with S10 for the times indicated. Samples were analyzed by Western blotting. (**E**) Time kinetics of three independently performed experiments are shown. Values represent the arithmetic mean ± SD.

Since every naturally occurring IDE-insensitive p6 analyzed so far possesses an N-terminal proline, we hypothesized that the introduction of Pro-1 could stabilize the IDE-sensitive p6 of HIV-1_NL4-3_. To challenge this hypothesis leucine at position one of pNL*env*1 p6 was replaced with proline resulting in the construct pNL*env*1 L1P. The corresponding *v*p6 was incubated with S10 for the times indicated in a steady state in vitro degradation assay and analyzed by Western blot. While p6 derived from pNL*env*1 *wt* is degraded in S10 as reported previously [16], p6 derived from pNL*env*1 L1P shows enhanced stability towards IDE-mediated degradation (Figure 2A). Vice versa, after replacement of the Pro-1 of the IDE-insensitive SG3 p6 with leucine, SG3 p6 loses its resistance to IDE (Figure 2B). As previously reported, kinetic analyses of *wt* p6 derived from pNL*env*1 incubated with S10 revealed a rapid, exponential decay with a half-life of about 5 min (Figure 2C,E). In contrast, the half-life of the pNL*env*1 L1P *v*p6 peptide is greater than 60 min. Vice versa, while SG3 *wt v*p6 shows no detectable degradation after 60 min, the mutant SG3 P1L *v*p6 has restored susceptibility to IDE and is degraded in S10 with a half-life of approximately 20 min (Figure 2D,E). Altogether, these results indicate that indeed the N-terminus of p6 regulates susceptibility of p6 to IDE-mediated degradation. Introduction of Pro-1 is sufficient to strongly enhance the stability of p6 that was previously a bona fide substrate for IDE [16].

**Figure 3:**
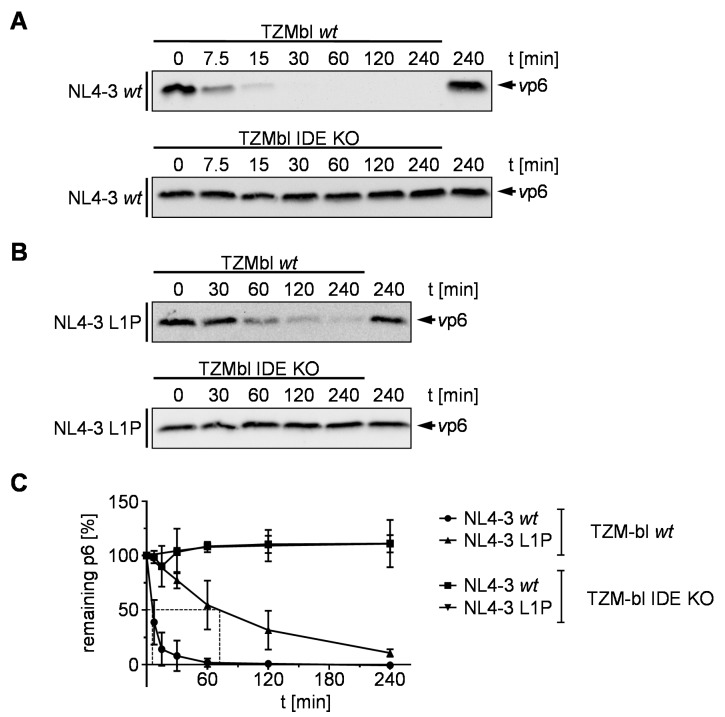
Longtime degradation kinetics of p6 derived from NL4-3 *wt* and L1P. NL4-3 *v*p6 derived from the mutants NL4-3 *wt* (**A**) and L1P (**B**) were incubated with S10 derived from TZMbl *wt* or IDE KO cells, respectively. After incubation for the times indicated, reactions were stopped by heat inactivation. Samples were analyzed by Western blotting using a p6-reactive antiserum. Representative Western blots of three independent experiments are shown. (**C**) Time kinetics of three independently performed experiments is shown. Values represent the arithmetic mean ± SD.

Proteolysis of the p6 L1P mutant is significantly reduced, but not completely impaired as shown by the in vitro degradation kinetic assay (Figure 2E). To compare t_1/2_ of p6 P1L and to examine whether the residual degradation of p6 is still IDE-mediated, we performed long-time degradation experiments with *v*p6 derived from NL4-3 *wt* and L1P in an S10 extract derived from HeLa TZMbl *wt* (S10 TZMbl *wt*) and IDE knock out cells (S10 TZMbl IDE KO). While *wt v*p6 is rapidly degraded in S10 TZMbl *wt* with a t_1/2_ of 5 min, comparable to S10 derived from HeLa SS6 cells, no degradation was detected after 4 h incubation of *wt v*p6 in S10 TZMbl IDE KO (Figure 3A,C). In contrast, the half-life of *v*p6 derived from NL4-3 L1P was increased to up to 70 min in S10 TZMbl *wt*. However, as in the case of *v*p6 derived from pNL*env*1 *wt,* no degradation was detected in S10 TZMbl IDE KO, indicating that the residual proteolysis of p6 L1P is IDE-mediated (Figure 3B,C).

Under membranous conditions, the HIV-1 p6 protein consists of two α-helices linked by a flexible domain [59]. Thereby, the N-terminus, containing the PTAP motif, should be unstructured. To investigate if the N-terminal proline residue affects the secondary structure of p6, structure prediction was conducted using the Phyre2 protein folding recognition server. The protein alignment of the predicted structures indicates that the Pro-1 within the sequence of NL4-3 p6 does not influence the structure of p6 (Figure S1A). Furthermore, for SG3 the prediction shows no major influence of the mutation P1L on the overall structure of p6 (Figure S1B).

### 3.3. Effect of the pNLenv1 L1P Mutation on Late Steps of the HIV-1 Replication Cycle

Previously, we were able to show that neither the artificial stabilization of p6 by triplication of the PTAP motif nor expression of HIV-1_NL4-3_ *wt* in IDE KO cells had any implication on Gag processing, viral budding or incorporation of Vpr [16]. However, according to previous reports, Gag cleavage-site mutants, like the mutant p6 L1F, can affect the efficiency of Gag processing by the viral protease [60]. Furthermore, a proline at position one of p6 is, in some cases, connected to certain mutations in the viral protease gene occurring under antiretroviral treatment [60,61]. The mutants described here, indeed, also showed increased amounts of NC-p6 in the steady state degradation assay (Figure 2A,B). Consequently, we wanted to quantify this delayed processing of p6 and investigate if the mutation p6 L1P has any further effects on the late steps of the viral replication cycle.

**Figure 4:**
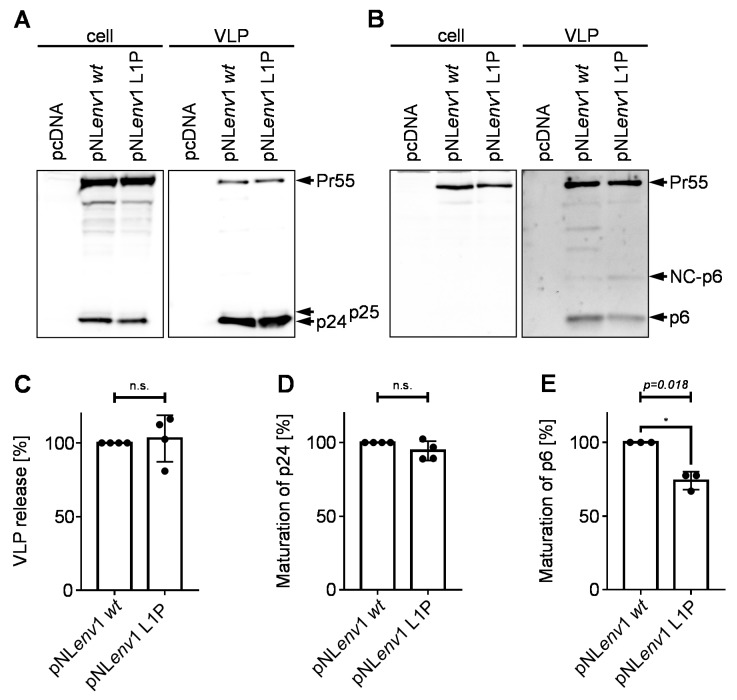
Influence of the mutation L1P on virus release and Gag-processing. HeLa SS6 cells were transfected with pNL*env*1 *wt* or pNL*env*1 L1P, respectively. Cell lysates and VLP fractions were analyzed by Western blotting using a p24-reactive (**A**) or a p6-reactive (**B**) antiserum. (**C**) The efficiency of virus release was calculated as the ratio of Gag (Pr55 and p24) present in the VLP fraction relative to the total amount of Gag detected in cells and released VLPs. (**D**) The rate of p24 processing was determined by calculating the ratio of p24 vs. Gag (Pr55 and p24) detected in released VLPs. (**E**) The rate of p6 processing was determined by calculating the ratio of p6 vs. Gag (Pr55, NC-p6, and p6) detected in released VLPs. (C–E) Band intensities were densitometrically quantified with AIDA. Values of pNL*env*1 *wt* were set to 100%. Scattered blots with columns representing mean values of four (C,D) or three (E) independent experiments ± SD. One sample *t*-test was conducted to determine statistically significant differences in virus release (C), p24 processing (D) and p6 processing (E) between the mutant pNL*env*1 L1P and pNL*env*1 *wt* (*p < 0.05; not significant (n.s.) *p* ≥ 0.05).

Therefore, HeLa SS6 cells were transiently transfected with pNL*env*1 *wt* or L1P, respectively. Western blot analysis of cell lysates and VLP fractions revealed that the mutant pNL*env*1 L1P does not affect the viral budding and maturation of p24 (Figure 4A,C,D). However, the L1P mutant exhibits a reduction in the maturation of p6 (Figure 4B,E). In line with this, the amount of NC-p6 is slightly increased. Furthermore, the mutant pNL*env*1 L1P lacks non-canonical Gag-cleavage products between NC-p6 and CA-NC-p6 that are present in VLPs derived from pNL*env*1 *wt* [62].

### 3.4. Stabilization by an N-Terminal Proline Renders HIV-1 Replication Resistant to Treatment with an IDE Inhibitor

Previously, we reported that stabilization of p6 via triplication of the PTAP motif results in reduced replication capacity of X4 tropic HIV-1 in PBMC. Furthermore, the IDE specific inhibitor 6bk reduces the X4-tropic replication capacity of HIV-1_NL4-3_ *wt*, while 6bk does not affect the mutant HIV-1_NL4-3_ 3xPTAPPA [16]. Although duplications of the PTAP motif are described to occur in patients under antiretroviral therapy (ART), the triplication of this motif is merely artificial. In contrast, HIV-1 p6 variants with an N-terminal proline occur as natural polymorphism in up to 20% of the isolates [37]. Hence, we wanted to investigate the influence of 6bk on the replication capacity of the natural occurring IDE-insensitive p6-variants.

**Figure 5:**
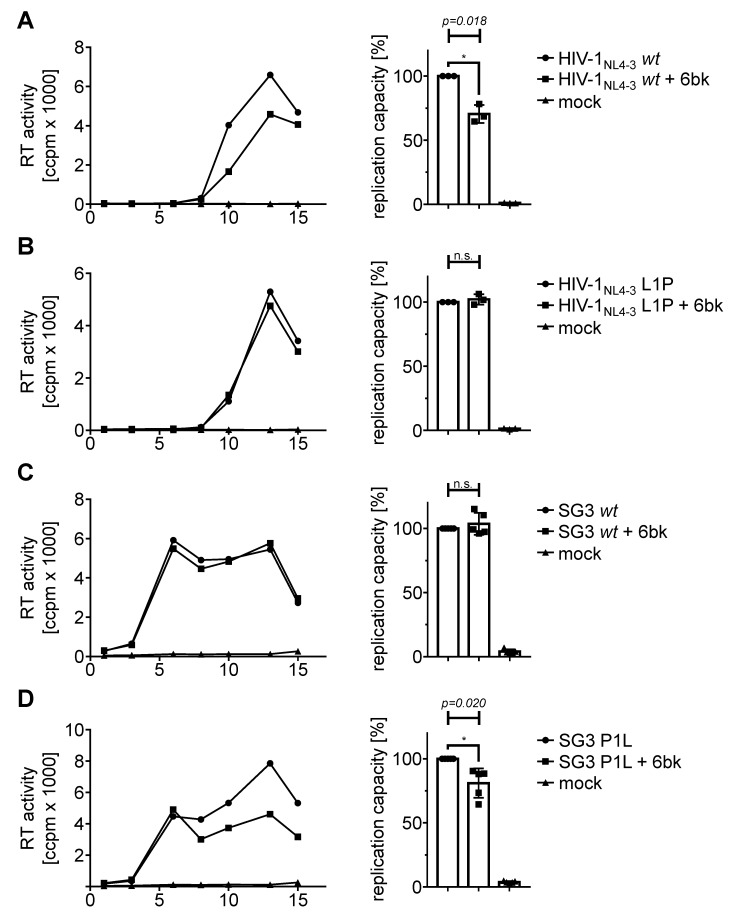
Influence of the IDE inhibitor 6bk on the replication capacities of X4-tropic HIV-1 NL4-3 L1P and SG3 P1L. Representative replication profiles are shown for infected PHA-IL2-stimulated PBMCs. Cells were infected with X4-tropic HIV-1_NL4-3_ *wt* (**A**), HIV-1_NL4-3_ L1P (**B**) (each 30 pg p24, MOI 10^−4^), SG3 *wt* (**C**) or SG3 P1L (**D**) (each 60 pg p24, MOI 10^−2^) with or without permanent treatment with 10 μM 6bk (left). Uninfected and untreated PBMCs served as mock control. Replication was assessed by quantification of the virus-associated RT activity contained in cell culture supernatant collected on the indicated dpi. The replication capacity of X4-tropic HIV-1_NL4-3_ wt, HIV-1_NL4-3_ L1P, SG3 *wt* or SG3 P1L following infection of PHA-IL2-stimulated PBMCs with and without permanent treatment with 10 µM 6bk was assessed by calculating the area under the curve (AUC) from each replication profile (right). The replication capacity of untreated cells in each experiment was set to 100%. Scattered blots with columns representing mean values of three (A,B) or five (C,D) independently performed experiments ± SD. One sample *t*-test was conducted to determine statistically significant differences between the replication capacity of treated and untreated cells (*p < 0.05; n.s. *p* ≥ 0.05).

Therefore, activated human PBMCs were infected with standardized amounts of X4-tropic HIV-1_NL4-3_ *wt* or L1P, respectively. Cell culture supernatants were collected on the indicated dpi and analyzed for release of virus particles by measuring the virus associated RT activity. For each virus, one representative replication profile is shown with and without treatment with 10 µM 6bk (Figure 5A–D left). Since the replication of HIV-1 in PBMCs generally exhibits donor-dependent differences, the area under the curve (AUC) representing the replication capacity in PBMCs was determined (Figure 5A–D right). Comparison of the replication profiles of HIV-1_NL4-3_ *wt* under treatment with 10 µM 6bk compared to the replication profiles of untreated PBMCs derived from three different donors reveals a 30% reduction in the HIV-1 replication capacity (Figure 5A). These results are consistent with previous reports [16]. However, by replacing just one leucine residue by proline in HIV-1_NL4-3_ p6 resulting in the mutant HIV-1_NL4-3_ L1P, X4-tropic replication of HIV-1 in activated PBMCs becomes utterly insensitive towards treatment with 10 µM 6bk (Figure 5B). Similar results have been observed with the IDE-insensitive p6 mutant 3xPTAPPA [16]. Thus, these data confirm that the reduction of the replication capacity of X4-tropic HIV-1_NL4-3_ by the IDE inhibitor 6bk in activated PBMCs is p6-dependent.

We also tested the influence of 6bk on the replication profile of SG3. Since SG3 harbors a naturally IDE-insensitive p6 variant, we hypothesized that replication should not respond to treatment with 6bk. In Figure 5C, a representative replication profile of SG3 is depicted in activated PBCMs with and without treatment with 10 µM 6bk. Consistent with our hypothesis, 6bk does not influence the replication of SG3 in PBMCs derived from five different donors. In contrast, the N-terminal mutation of the proline residue, resulting in an IDE-sensitive variant, leads to a reduction in the replication capacity by up to 20% following treatment with 6bk (Figure 5D).

### 3.5. Phylogenetic Background of the N-Terminus of p6

Based on the results obtained with the HIV-1 isolates SG3 and 4lig7, we were able to identify one single aa substitution that regulates susceptibility of p6 towards IDE-mediated degradation. Since the N-terminal proline exhibits a naturally occurring polymorphism, we investigated the phylogenetic background of the p6 N-terminus.

**Figure 6:**
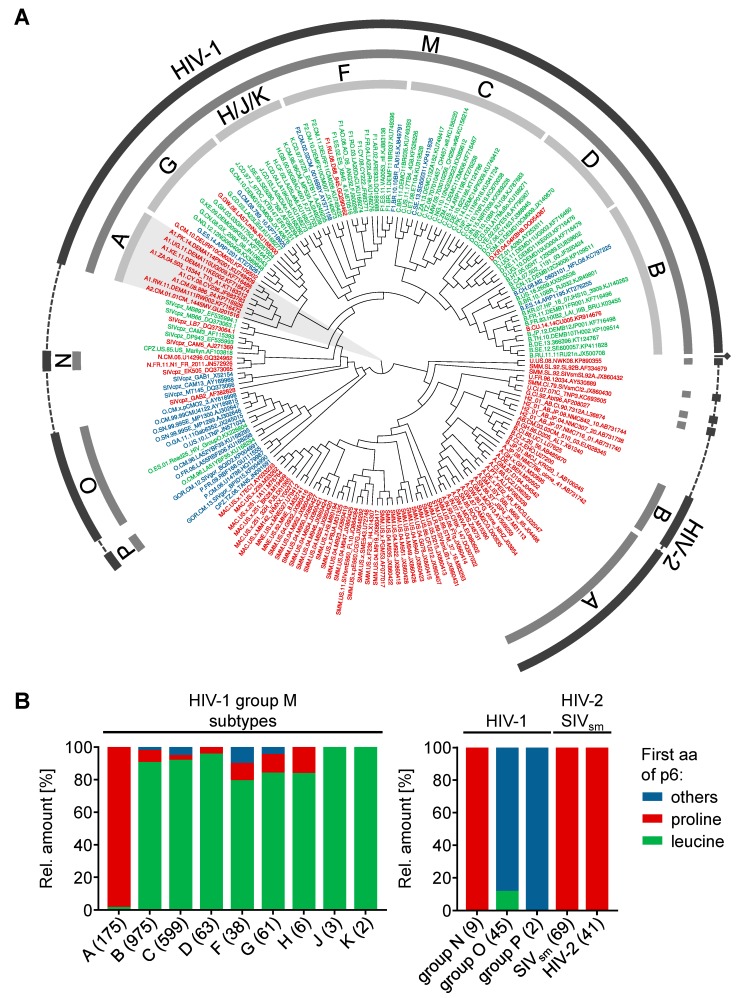
Phylogenetic background of the aa at position one of p6. (**A**) SATé HIV-1/SIV/HIV-2 phylogeny in circular form. In the phylogenetic tree, the isolates have been colored corresponding to the first aa of p6. Isolates with a proline at position one of p6 are depicted in red, isolates in which p6 begins with leucine are colored green, and all other isolates are written in blue. The labeled rings indicate the virus species HIV-1 or HIV-2, respectively (outer), groups (middle) and subtypes (inner). (**B**) A total of 2088 representative sequences from the Los Alamos Sequence Database have been analyzed in silico regarding the first aa of p6. Depicted are the aa occurrences at position one of the p6 peptides of all HIV-1 groups and subtypes, SIVsm (including SIVmac sequences) and HIV-2. Sequences of p6, which begin neither with proline (red) nor with leucine (green), have been summarized as others (blue). The number of analyzed sequences is indicated in brackets and was set to 100% for each column.

To gain a better understanding of the phylogenetic relationships of the different subtypes in the context of the N-terminus of p6, we constructed a midpoint rooted, polar cladogram of HIV-1, several SIV sequences and HIV-2 using complete genome sequences. Several SIVcpz isolates derived from chimpanzees of the subspecies *Pan troglodytes troglodytes* that are closely related to HIV-1 have been included in the phylogenetic tree [31,63]. Withal, we colored the labeling of the isolates corresponding to the first aa of p6. Depicted in green are isolates with a p6 peptide beginning with a leucine residue, red for isolates in which p6 starts with a proline residue or blue for p6 proteins that have neither a leucine residue nor a proline residue at their N-terminus (Figure 6A).

In contrast to HIV-1, HIV-2 is not categorized in distinct subtypes, but several groups have been identified, each most likely originating from a separate species transmission of SIV to humans [64]. However, neither the p6 derived from different HIV-2 groups nor p6 derived from any other SIVsm isolate contains an N-terminus that is different from proline.

In the clade of HIV-1, the groups O and P are the most distantly related groups to the pandemic HIV-1 group M. In contrast, the HIV-1 group N is more closely related to HIV-1 group M. However, the HIV-1 groups O and P are the most heterogenic groups regarding the N-terminus of p6, whereas the p6 protein of HIV-1 group N exclusively begins with an N-terminal proline.

Surprisingly, the predominant N-terminal leucine residue is a characteristic of HIV-1 group M. Within HIV-1 group M, the clade containing the subtypes A and G split early from the other subtypes. Intriguingly, while all subtypes in the phylogenetic tree share the characteristic of a predominant leucine residue at the N-terminus of p6, subtype A exclusively expresses p6 proteins with an N-terminal proline. Within the viruses analyzed herein, some SIVcpz isolates are positioned near the clade of HIV-1 group M. Concerning p6, two of these three isolates, MB66 and MB897, begin with leucine, while the isolate LB7 has an N-terminal proline [31,65]. Our phylogenetic analysis indicates that the N-terminal leucine of p6 formed in the SIVcpz population even before the pandemic HIV-1 group M was established in humans.

Except subtype A, the HIV-1 group M is primarily characterized by a p6 protein that begins with an N-terminal leucine. However, although the Los Alamos sequence compendium is designed to provide a representative overview of known HIV-1 and HIV-2 sequences, the manual selection might bias the actual aa occurrences of the different isolates [48,49]. To gain a more accurate quantification of the discrepancy between subtype A and the other subtypes and groups, we analyzed a total of 2088 sequences derived from HIV-1, SIVsm and HIV-2 in silico regarding the aa at position one of p6 [54]. Depicted in Figure 6B is the ratio of p6 sequences beginning with a leucine residue (green), a proline residue (red) or another aa (blue). As already indicated by the phylogenetic tree, the p6 protein derived from HIV-2 and SIVsm sequences is consistent regarding the N-terminal aa. Here, all analyzed p6 sequences code for a p6 protein that begins with a proline, without any exception. Similarly, the HIV-1 group N is the only group, in which proline is the only aa at the N-terminus of p6. In contrast, none of the p6 proteins of group O have an N-terminal proline, and only 11.1% of the isolates of group O have an N-terminal leucine residue in p6.

Around 80% to 100% of the p6 sequences derived from the HIV-1 group M subtypes B-K begin with an N-terminal leucine. In contrast, proline is only the second most common aa at position one of mature p6 in group M, with 14.5% of all sequences. For subtype B, the subtype of HIV-1_NL4-3_, SG3, and 4lig7, only 7.4% of the isolates contain p6 sequences with an N-terminal proline, whereas 90.1% of the sequences have an N-terminal leucine residue. In contrast, the HIV-1 subtype A is the only exception for this characteristic N-terminus of p6. Here, 98.9% of all available p6 sequences begin with an N-terminal proline. It should be mentioned that the total amount of available sequences for each group and subtype are highly variable.

### 3.6. IDE-Susceptibility of p6 Derived from Various Subtypes

The phylogenetic analysis of the N-terminal aa of HIV-1 group M revealed that the proline at position one of p6 is a particularity of subtype A. Since the proline at the N-terminus of p6 renders p6 IDE-insensitive, we hypothesized that p6 peptides from subtype A viruses are not degraded by IDE. To investigate this hypothesis, we infected CEMx174 M7 R5 cells with several HIV-1 field isolates, representative of different HIV-1 subtypes. After syncytia formation or five dpi, we collected the cell culture supernatants and isolated the viral particles. After the lysis of the viral particles, we performed a steady state in vitro degradation assay with rIDE.

**Figure 7:**
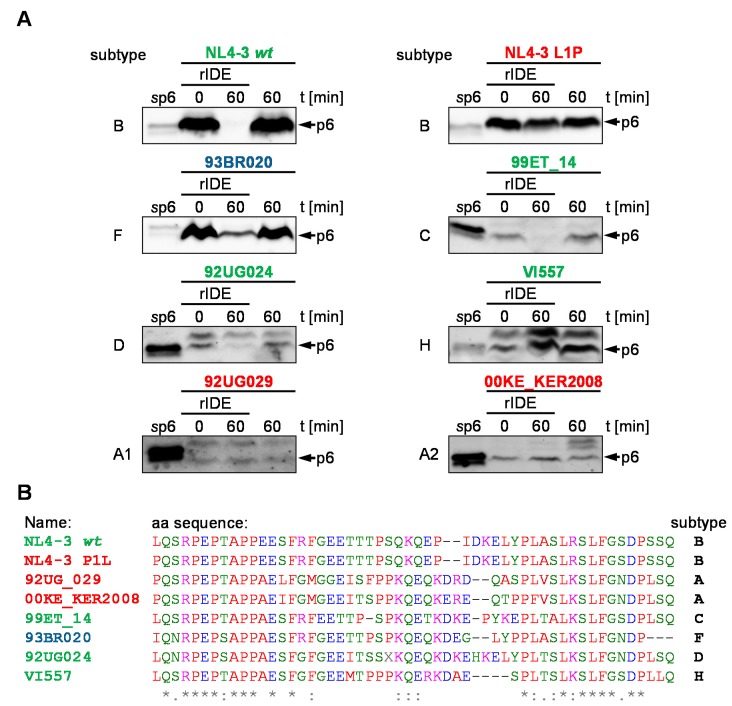
Degradation of p6 derived from various subtypes by IDE. (**A**) rIDE was incubated with *v*p6 derived from the indicated field isolates. Reactions were stopped by heat inactivation either immediately or after incubation for 60 min at 37 °C. p6 incubated with IDE buffer for 60 min at 37 °C served as negative control. Representative Western blots of four independent experiments are shown. (**B**) Sequence alignment of the isolates tested for sensitivity towards IDE-mediated degradation. Colors of the sequence alignments according to the physicochemical properties of the aa, as proposed by the Clustal Omega multiple sequence alignment software. Conserved aa positions in the sequence alignment are indicated by asterisks. Furthermore, aa exchanges between strongly (colon) and weakly (dot) similar aa residues are specified [45,46,47].

While most non-A isolates are degraded by rIDE within 60 min at 37 °C, no degradation was detected for both HIV-1 subtype A isolates 92UG029 and 00KE_KER2008, respectively (Figure 7A). Both subtype A isolates have a proline at the N-terminus of p6. In contrast, even the subtype F isolate 93BR020, harboring a p6 sequence with an N-terminal isoleucine residue is degraded by IDE (Figure 7A,C). However, the subtype F 93BR020 and the subtype D isolate 92UG024 might differ in their degradation kinetics. Here, a faint band is still detectable after 60 min degradation, indicating that there have been further adaptions to IDE-mediated degradation in different isolates. Furthermore, p6 derived from the subtype H isolate HIV-1 VI557 is not degraded by rIDE (Figure 7A). Intriguingly, the isolates 92UG024 (subtype D), VI557 (subtype H), and 92UG029 (subtype A) show a band slightly larger than p6 which also is reactive with our polyclonal p6 Ab and which is not degraded by IDE. In our experiments, subtype A correlates with the stability of p6 towards IDE-mediated degradation. Intriguingly, the IDE-insensitive p6 variant of the subtype H isolate VI557 is, of all analyzed isolates, the closest relative to the analyzed subtype A isolates.

## 4. Discussion

The HIV-1 p6 protein is rapidly degraded by IDE. This phenomenon is specific for HIV-1 since p6 from HIV-2 ROD10 and SIVmac239 proved to be IDE-insensitive [16]. Although proteolysis of p6 by IDE appears to be a common feature of HIV-1, the phylogenetic origin of the degradation of p6 remained unclear until now [16]. Here, we describe two p6 sequences of HIV-1 that are not degraded by IDE: One originates from the molecular clone SG3, the other from the multiresistant recombinant virus 4lig7 [40]. These isolates are part of the HIV-1 group M subtype B, the most common subtype in the United States [66]. Intriguingly, both p6 sequences begin with an N-terminal proline, an attribute they have in common with the IDE-insensitive p6 peptides from HIV-2 and SIV as well as the EIAV p9 protein [16]. Thus, it was legitimate to hypothesize that the N-terminal proline regulates the susceptibility of p6 to IDE-mediated degradation.

To elaborate the role of an N-terminal proline in IDE-mediated degradation of p6, we mutated the first aa of p6 from HIV-1_NL4-3_ derived pNL*env*1 *wt* to proline, resulting in the construct pNL*env*1 L1P. Accordingly to our hypothesis, p6 derived from pNL*env*1 L1P has an increased half-life compared to p6 *wt.* Furthermore, the reciprocal mutation of SG3 *wt* to SG3 P1L resulted in a p6 variant with restored susceptibility to IDE-mediated degradation. These results indicate that the N-terminus of p6 is of importance for the degradation of p6 by IDE. This finding is supported by observations of Shen et al., who found that the N-terminus of substrates is vital for substrate-binding to IDE. The first 3–5 aa of an IDE substrate, with the cleavage-site containing 7–13 aa, form a β-sheet together with the IDE strands β12 and β6 [17]. Thus, an N-terminal proline might interfere with this β-sheet, thereby preventing substrate-binding of p6 to IDE.

Previously, we described an IDE-insensitive p6 mutant bearing a triplication of the PTAPPA motif (p6 3xPTAPPA). We assumed that the triplication of the PTAPPA motif increases the length of p6 beyond the size-exclusion limit of the IDE. However, due to a duplication of its N-terminus, the p6 protein from HIV-1 IIIB is of similar size as the p6 3xPTAPPA. Albeit, p6 derived from HIV-1 IIIB is susceptible to degradation in S10 extract, contradicting the size-exclusion of the p6 3xPTAPPA [16]. The 3xPTAPPA is adjacent to an N-terminal cleavage site. Thus, the proline-rich PTAPPA motif near the major N-terminal IDE cleavage site might also prevent the formation of the β-sheets with IDE strands β12 and β6, necessary for binding to IDE [17]. Consistent with this, the p6 3xPTAPPA mutant fails to compete with *wt* p6 for degradation by IDE, thus indicating that it cannot bind to IDE [16].

Regarding the role of p6 in late processes, the mutant pNL*env*1 L1P shows *wt* phenotype. However, maturation of p6 is slightly impaired compared to *wt* due to the mutation of the p1/p6 cleavage site. Previously, the Gag cleavage site between p1 and p6 has been associated with several protease mutations [60,61]. Particularly, a proline at the N-terminus of p6 has been associated with treatment failure in HIV-1 positive patients. Thereby, this aa has been statistically associated with the protease inhibitor (PI) resistance mutations K20I/R/M and L89M/I within the PR gene [67]. Consistent with this, the IDE-insensitive multiresistant field isolate 4lig7 bears the PR mutation K20I alongside other resistance mutations. Since the p6 protein of the mutant pNL*env*1 L1P shows altered processing by the viral PR compared to pNL*env*1 *wt*, a natural L1P adaption in the p6 gene might occur to compensate a PI resistance mutation in the protease, restoring viral fitness. However, the correlation of the N-terminal proline to PI treatment and resistance mutations in PR is still part of an ongoing debate. Verheyen et al. describe the N-terminal proline of p6 as natural polymorphism, without any correlation to PI resistance mutations [37]. Furthermore, no correlation of p6 L1P to mutations in PR has been described in the Swiss HIV-1 cohort [68]. Intriguingly, in this cohort, the N-terminal proline is associated with three other mutations, namely an HIV-1 p6 S3G substitution and the mutation HIV-1 p6 P5L/T [68]. In the case of the formation of a β-sheet of p6 with IDE, all three mutations might be involved in the binding to IDE [17].

Duplication of the PTAP motif frequently develops under antiretroviral therapy [69,70,71]. In contrast, the previously described IDE-resistant p6 mutant “3xPTAPPA” is merely artificial in as much as triplications of the PTAP motif have not been found to arise naturally [16]. Thus, it has not been possible to follow potential effects of stable p6 on the HIV-1 pathology in vivo. However, the natural p6 polymorphism Pro-1 will now allow, for the first time, to investigate the biological functions of IDE-mediated degradation of p6 in vivo. Thereby, stabilization of p6 by a naturally occurring N-terminal proline does not only allow performing intersubtype evaluations, but also enables evaluation of the IDE-dependent HIV-1 progression of patients infected with the same subtype. Subtype B is the most prevalent in HIV-1 isolates and accounts for 66% of all global HIV-1 infections [72]. In future studies, it might be interesting to investigate if there are differences in the progression of HIV-1 pathology between the 7.4% of the patients infected with subtype B coding for a p6 protein with an N-terminal proline, and patients infected with a subtype B containing an IDE-susceptible p6. The identification of one single aa substitution that regulates IDE susceptibility of p6 allowed us to investigate the phylogenetic background of this natural polymorphism.

Interestingly, all described HIV-2 and SIVsm isolates have an N-terminal proline in their p6 sequence indicating a general stability of HIV-2/SIVsm p6, not only for the described p6 variants from HIV-2 ROD10 and SIVmac239 [16]. Towards HIV-1, the N-terminus of p6 becomes much more polymorphic, indicating that the selection pressure in the recent evolutionary history of HIV-1 favored more heterogenic N-termini for p6. The N-terminus of p6 derived from group O is genetically the most heterogenic, while p6 from the HIV-1 group N exclusively consists of p6 with an N-terminal proline. The HIV-1 group M is the only group of HIV-1 that predominantly consists of p6 variants with an N-terminal leucine. However, the N-terminal leucine is also present in SIVcpz isolates closely related to HIV-1 group M [31,63]. In contrast to the other subtypes, nearly all isolates of HIV-1 subtype A have a proline at their N-terminus. A similar result has been observed previously by Torrecilla and colleagues, who observed the conservation of PR cleavage sites among different subtypes [73]. Intriguingly, the proline at the N-terminus of p6 in subtype A is genetically exceptionally stable and occurs in nearly all HIV-1 subtype A isolates described. Furthermore, the cleavage site between p1 and p6 is significantly more conserved within subtype A compared to the other subtypes [73,74]. If the transmitted virus that led to the formation of the HIV-1 group M had a p6 sequence with an N-terminal leucine, then the subtype A achieved the N-terminal proline subsequently to the transmission of HIV-1 from chimpanzees to humans. According to Patiño-Galindo et al., the most recent common ancestor of HIV-1 subtype A1 dates back to around the year 1949, being the earliest most recent common ancestor described for all HIV-1 subtypes [75]. However, the phylogenetic data do not allow it to be said with certainty that the virus transmitted to humans had leucine at the first position of p6, and another possibility would be that the first HIV-1 group M virus had an N-terminal proline. Consequently, this would mean that the predominant leucine residue formed at least two times independently within HIV-1 group M. The first time, when HIV-1 subtype G split from subtype A, and some other time when the other subtypes split from subtype A and G.

Although it has not a defined recognition motif, IDE is reported to be highly selective. Thus, it degrades glucagon, while it spares related peptides like the glucagon-like peptide or the glucagon fragment glucagon_19-29_ [76,77,78]. Our data suggest that susceptibility of HIV-1 p6 to IDE-mediated degradation is entangled with the aa at the N-terminus of p6. However, while a proline at aa position one of p6 prevents degradation of p6 by IDE, we cannot deduce as a rule that an N-terminal leucine renders p6 susceptible to IDE-mediated degradation. Thus, the p6 protein derived from the subtype H isolate VI557 is not degraded by rIDE, although it has an N-terminal leucine. Since several adaptions can modify the susceptibility of p6 to IDE, as well as the high degradation rate of p6, this hints towards a selective interaction of p6 and IDE.

Previously, we have shown that the reduced replication capacity of HIV-1 after stabilization of p6 is *env*-dependent. While R5-tropic HIV-1 does not respond to stabilization of p6, replication of X4-tropic HIV-1 is reduced under the same conditions in vitro [16]. Intriguingly, the HIV-1 subtype A bearing IDE-insensitive p6 variants is more likely to be R5-tropic than subtype D in non-AIDS patients [79]. Furthermore, we hypothesized that p6 might partially compete with the clearance of amyloid β in the brain, thereby contributing to the progression of HIV-1 associated neurocognitive disorders [16]. This is supported by the finding that, in comparison to HIV-1 subtype D, patients infected with a subtype A virus have a lower risk to develop a cognitive impairment [80].

If stabilization of IDE substrates by an N-terminal Pro-1 is possible in principle, this might also be of interest to increase the half-life of insulin preparations and thereby to enhance bioavailability of administered insulin in diabetes patients. Furthermore, a potential role of IDE in the development of diabetes and Alzheimer’s disease might be addressed using insulin or amyloid-β peptide, respectively, that is not degraded by IDE but has a normal susceptibility to other degradation pathways [81,82,83,84]. However, it must be clarified whether stabilization of IDE substrates, other than p6, by mutation of their N-terminus to proline is feasible and does not affect the processing of their precursor protein [85,86].

Stabilization of p6 by treatment with the IDE-specific inhibitor 6bk, or the infection with HIV-1 harboring an IDE-resistant p6 variant resulted both in a slightly but statistically significant reduction of the replication capacity. Simultaneously, all known functions of p6 described so far are not affected. This, altogether, hints towards a completely unknown function of mature p6 [16]. Besides the replication capacity, the p6 protein might affect other aspects of the HIV-1 infection that are not represented in our cell culture systems, as it has been shown previously for several accessory HIV proteins [87].

## 5. Conclusions

Overall, our data indicate that a proline residue at the N-terminus of p6 prevents the degradation of the HIV-1 p6 protein by IDE. Intriguingly, the Pro-1 is characteristic for HIV-1 subtype A. Consequently, IDE does not degrade p6 proteins derived from isolates of this subtype. In contrast, susceptibility of p6 to IDE-mediated degradation is conserved in at least four other subtypes of HIV-1.

## Supplementary Materials

The following is available online at http://www.mdpi.com/1999-4915/10/12/710/s1, Figure S1: Mutation of the N-terminal aa does not affect the predicted secondary structure of p6.
