# Supplementary material for: The N-Terminus of the HIV-1 p6 Gag Protein Regulates Susceptibility to Degradation by IDE
Source: Viruses. 2018 Dec 12;10(12):710. doi: 10.3390/v10120710 (PMC6316412; doi:10.3390/v10120710)
Supplement: Supplementary file 1 [file viruses-10-00710-s001.pdf]

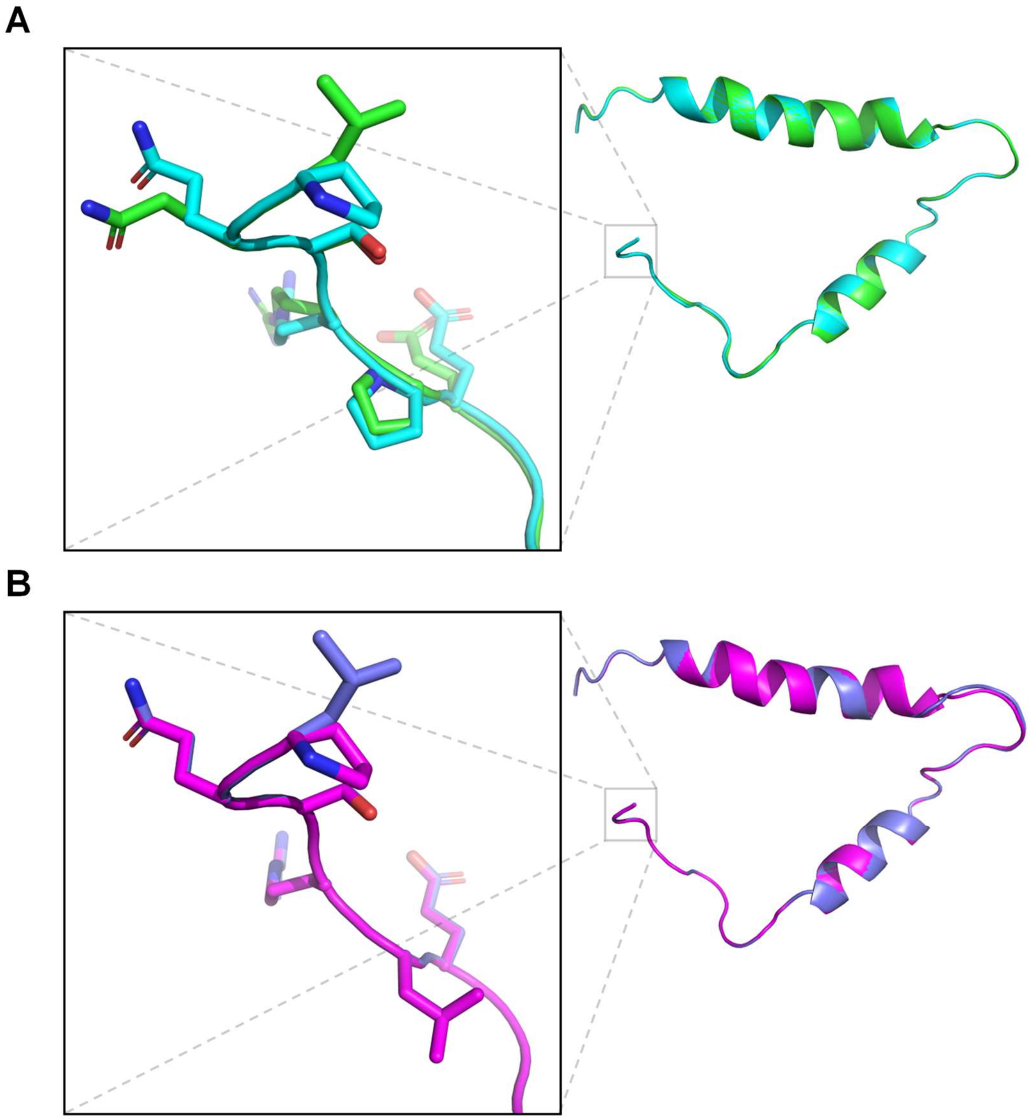

Figure S1. Mutation of the N-terminal aa does not affect the predicted secondary structure of p6. Structure alignment of p6 derived from HIV-1NL4-3 wt (green) and L1P (light blue) (**A**) or SG3 wt (purple blue) and P1L (magenta) (**B**) as predicted using the Phyre2 protein folding recognition server. A close up of the first six aa residues is shown as an inset.
